# Signal processing of functional NIRS data acquired during overt speaking

**DOI:** 10.1117/1.NPh.4.4.041409

**Published:** 2017-09-11

**Authors:** Xian Zhang, Jack Adam Noah, Swethasri Dravida, Joy Hirsch

**Affiliations:** a Yale School of Medicine, Department of Psychiatry, New Haven, Connecticut, United States; b Yale School of Medicine, Interdepartmental Neuroscience Program, New Haven, Connecticut, United States; c Yale School of Medicine, Department of Neuroscience, New Haven, Connecticut, United States; d Yale School of Medicine, Department of Comparative Medicine, New Haven, Connecticut, United States; e University College London, Department of Medical Physics and Biomedical Engineering, London, United Kingdom

**Keywords:** near-infrared spectroscopy, functional NIRS, functional neuroimaging, speech production, principal component analysis, spatial filter

## Abstract

Functional near-infrared spectroscopy (fNIRS) offers an advantage over traditional functional imaging methods [such as functional magnetic resonance imaging (fMRI)] by allowing participants to move and speak relatively freely. However, neuroimaging while actively speaking has proven to be particularly challenging due to the systemic artifacts that tend to be located in the critical brain areas. To overcome these limitations and enhance the utility of fNIRS, we describe methods for investigating cortical activity during spoken language tasks through refinement of deoxyhemoglobin (deoxyHb) signals with principal component analysis (PCA) spatial filtering to remove global components. We studied overt picture naming and compared oxyhemoglobin (oxyHb) and deoxyHb signals with and without global component removal using general linear model approaches. Activity in Broca’s region and supplementary motor cortex was observed only when the filter was applied to the deoxyHb signal and was shown to be spatially comparable to fMRI data acquired using a similar task and to meta-analysis data. oxyHb signals did not yield expected activity in Broca’s region with or without global component removal. This study demonstrates the utility of a PCA spatial filter on the deoxyHb signal in revealing neural activity related to a spoken language task and extends applications of fNIRS to natural and ecologically valid conditions.

## 1 Introduction

Speech is a primary human function; however, brain activity related to tasks using overt speaking is difficult to investigate using traditional imaging methods, such as functional magnetic resonance imaging (fMRI), due to motion artifacts resulting from mouth and head movements. Language production has primarily been studied using imagined (covert or internal) speech^1^ or sparse sampling methods.^2^^,^^3^ These studies generally support classic literature on the canonical language system,^4^^–^^6^ in which brain activity associated with speech production has been localized to Broca’s region and supplementary motor cortex. This prior literature plus the gold-standard from lesion studies and neurosurgical interventions where cortical stimulations document functional loci for speech production based on picture-naming tasks^7^ provide a valid reference for the findings of this study. Our primary goal in this study was to develop a technique to reliably acquire hemodynamic signals during overt speech production. Here, we compare the blood oxygen level-dependent signals of fMRI using the picture-naming task and other prior language studies using Neurosynth^8^ with hemodynamic signals of functional near-infrared spectroscopy (fNIRS) (acquired during covert object naming) based on concentrations of both oxyHb and deoxyHb with and without spatial filtering.

Although fNIRS has been available as a neuroimaging methodology for more than 20 years,^9^^,^^10^^–^^15^ many technical and computational challenges remain in order to investigate spatially localized neural cognitive functions in adult subjects.^16^^–^^18^ However, one of the primary advantages of fNIRS includes signal acquisition in natural conditions that allow relatively free movement and communication. One of the specific challenges for this application includes filtering of systemic artifacts, such as effects of blood pressure and respiratory changes, that are often prominent in fNIRS signals.^16^^,^^19^^,^^20^ Overt speaking tasks, as compared to nonverbal cognitive tasks such as mental arithmetic, have been shown to effect breathing and the end-tidal ${\mathrm{CO}}_{2}$ concentration in blood (${\mathrm{PetCO}}_{2}$) with differential global effects on task-related changes in oxyHb and deoxyHb signals.^20^ The complex combination of effects due to speaking and breathing activities as well as volitional cognitive tasks challenges interpretations of fNIRS signals. In this paper, we attempt to address the issue of global systemic artifact using a spatial component removal method^21^ and using the deoxyhemoglobin (deoxyHb) signal, which may be less susceptible to global systemic components as well as local variations within and across subjects. However, both deoxyHb and oxyHb signals are shown for illustrative purposes.

The global systemic artifact in fNIRS is often addressed by using short channel recording,^22^^,^^23^ which is assumed to be only sensitive to systemic components that can be removed from the data. This approach is a method of choice for region-of-interest (ROI) studies that do not employ full head coverage. However, since short channel separation relies on the temporal characteristics of the waveform of the systemic artifact, this method is challenged by the fact that these artifacts can have similar waveforms to the task-related fNIRS signal.^16^^,^^21^^,^^22^ Thus, a regression method using temporal domain information from the short channels may remove both the global effects as well as the spatially localized task-related neuronal signals, reducing sensitivity to main effects.

To address this problem, we previously reported the results of a principal component analysis (PCA) spatial filter that was used to remove global components from oxyhemoglobin (oxyHb) and deoxyHb signals during a finger-thumb tapping task, with optode coverage that was distributed over most of the head.^21^ The effects of global systemic artifacts within the oxyHb signal were more pronounced relative to the deoxyHb signal. However, following the application of the PCA filter, the oxyHb signal also showed expected spatial specificity as did deoxyHb signals.

**Fig. 1:**
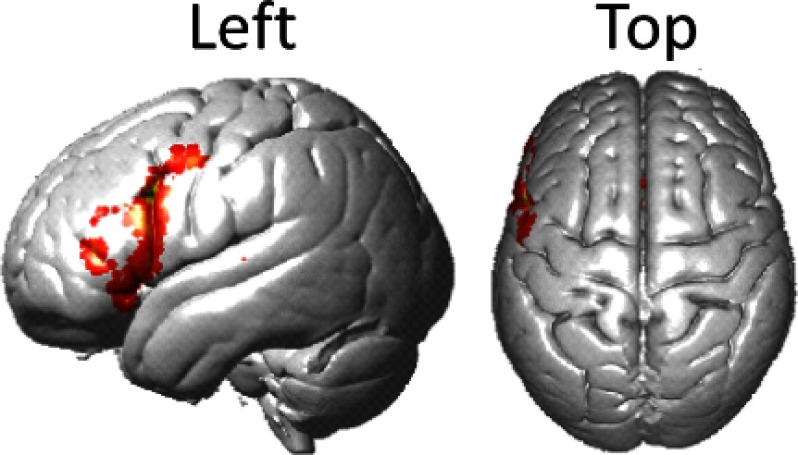
Neural activity determined by Neurosynth (meta-analysis of 6983 studies identified by the search term “Broca”) serves to identify one determination of the fiducial location of Broca’s area, the ROI for this investigation.

In this study, we applied the previously developed PCA spatial filter to fNIRS signals recorded during an overt picture-naming task, which was similar to the classic Boston Naming Test.^24^ In addition, we compared recorded fNIRS signals with fMRI data previously acquired during silent speech^25^ to evaluate the spatial correlation of results between these two methods using similar tasks and paradigms. Tasks that elicit hemodynamic signals with well-defined functional patterns, such as finger-thumb tapping or flashing checkerboard viewing, have typically been used to develop and verify fNIRS recording and systemic artifact removal techniques. Spatial patterns generated by simple language tasks, such as picture naming and description, can also be compared to meta-analyses of functional imaging results. Figure 1 shows the results of a Neurosynth forward inference map generated from a meta-analysis of 6983 studies using the search term “Broca.” Neurosynth is an online meta-analysis tool that uses references to specific terms in many published studies to generate activity maps.^8^ To generate the forward inference map, a statistical analysis is performed using the coordinates reported in studies that do and do not reference Broca’s region.

We employed picture naming and description in order to confirm well-known, previously verified, functional results that serve as fiducial markers for verification of the spatial filter technique. We aim to compare results from oxyHb and deoxyHb signals and two signal processing methods (with and without spatial filtering) to validate mapping procedures associated with spoken language using fNIRS.

## 2 Methods

### 2.1 Participants

A total of 22 individuals (14 female, mean age=24.5±7.8, ranging from 18 to 55 years) participated in the experiment. All were fluent English speakers but language history and lateralization was not obtained for this study. All but two participants were right-handed, as determined by the Edinburgh Handedness Inventory.^26^ No participants were excluded from the experiment. Written informed consent was obtained from each participant in accordance with guidelines approved by Yale University Human Investigations Committee (HIC #1501015178). All data were obtained from the Brain Function Laboratory at Yale School of Medicine, New Haven, Connecticut, and each person was compensated for their participation in the study.

### 2.2 Functional NIRS Signal Acquisition

**Fig. 2:**
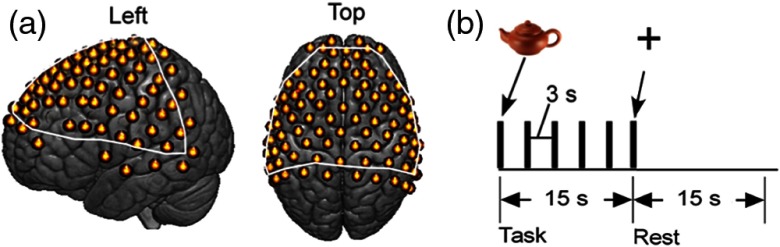
(a) 98-channel layout, covering frontal, temporal, and parietal lobes. The white outline in (a) represents the field of view reliably covered for all subjects in the fNIRS recordings. (b) Task paradigm: in each task block, five pictures were presented for 3 s each, which was followed by a 15-s rest block. Each run consisted of six task/rest cycles.

fNIRS signals were acquired using a LABNIRS system (Shimadzu Corp., Kyoto, Japan). Thirty emitter and 29 detector optodes were positioned 3 cm apart, providing a grid of 98 acquisition channels [Fig. 2(a)]. Each emitter optode connected to laser diodes at three wavelengths (780, 805, and 830 nm) used to measure changes in concentration of deoxyHb and oxyHb. Signals were acquired every 0.093 s. For analysis, signals were down-sampled to 0.93  samples/s by averaging 10 data points into one value.

### 2.3 Task and Paradigm

To investigate cortical activity during language production acquired by fNIRS, we used an overt picture-naming task that was similar to the object-naming tasks commonly used in fMRI for neurosurgical planning applications.^7^ Participants were instructed to name and give a short description of each picture, which was presented for 3 s. A 15-s task block (five pictures) alternated with a 15-s rest block [Fig. 2(b)]. Each run consisted of six task/rest cycles, and two runs were performed for a total of 6 min.

### 2.4 Optode Localization and Definition of Region of Interest

**Fig. 3:**
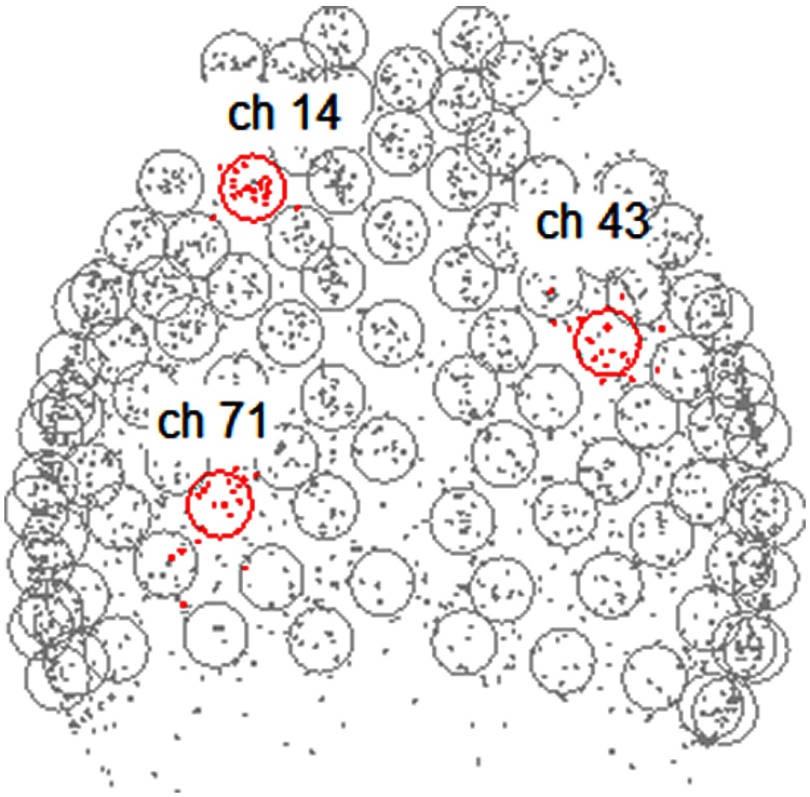
Channel location variability. Variability of channel locations across different participants is shown with a top-down projection view of all channels and subjects. Each circle is centered on the group median location of a channel. Each dot indicates the location of a channel for an individual participant. Locations for three exemplar channels, 14, 43, and 71, are shown in red. For example, each of the red dots around channel 71 represents the location of channel 71 for each individual participant.

The locations of emitters and receivers, along with standard 10 to 20 (Ref. 27) landmarks, including inion, nasion, Cz, T3, and T4, were determined using a Patriot three-dimensional (3-D) digitizer (Polhemus, Vermont). The Montreal Neurological Institute (MNI) coordinates for each recording channel and the corresponding anatomical locations of these channels were determined with the statistical parametric mapping package, NIRS-SPM.^28^ The native form of fNIRS data is channel-based since signals are recorded through channels and not individual voxels, which are interpolated between channel locations. Due to individual anatomical variations (e.g., head size and shape), the channel locations (represented by MNI coordinates) are not necessarily identical across participants (Fig. 3). To correct for these variations, we projected the data from each participant onto regions that represent the median channel locations for the group (Table 1 in Appendix A).

### 2.5 Functional NIRS Data Preprocessing

Temporal baseline drift was removed with the wavelet detrending algorithm procedure provided in NIRS-SPM.^28^ Global components were removed using the PCA spatial filter algorithm reported previously.^21^ The value of the width at half-maximum of the spatial filter was set at 46 deg rather than 50 deg. See Appendix B for a detailed explanation on the optimization of this parameter. Beta values (i.e., the amplitude of neural activity defined as the scale of best fit hemodynamic response function) were projected into MNI standard brain space ($2\times 2\times 2\;{\mathrm{mm}}^{3}$). Transforming fNIRS data into a 3-D volume is done with triangulation-based linear interpolation (using the grid data command in MATLAB). For voxels located directly on a channel, the spatial smoothing range was zero. For a voxel at the center of a triangular pyramid, the smoothing value was the mean of surrounding channels. In general, the range of spatial smoothing was less than 1.5 cm, half the distance between two channels. No additional smoothing was applied.

### 2.6 Voxel-Wise Analysis

First-level (single subject) and second-level (group) general linear model analyses were performed using SPM8.^29^ Beta values (i.e., hemodynamic signal amplitude as fit to the hemodynamic response function) were projected into MNI standard brain space using linear interpolation. Any voxel located farther than 18 mm away from the brain surface was excluded. In order to compare the effect of the task on the deoxyHb and the oxyHb signals, we have adopted a convention of inverting the polarity of the deoxyHb signals for the group analyses so that both oxyHb and deoxyHb data show the same polarity in terms of representing neural activity. A reduction in deoxyHb concentration and an increase in oxyHb concentration both correspond to “positive” fNIRS activity as represented by the figures and the reverse was true for “negative” activity. Results for the contrast, object naming versus rest, were rendered at threshold level p<0.05 corrected by a false discovery rate (FDR).^30^

## 3 Results

### 3.1 Deoxyhemoglobin

**Fig. 4:**
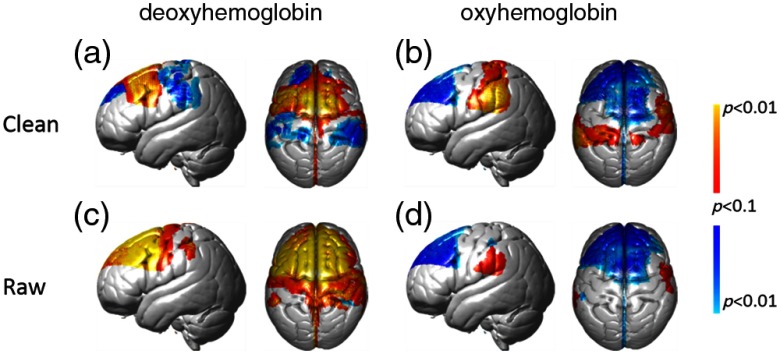
fNIRS results. fNIRS activity is shown with and without the global component removed at a lenient uncorrected threshold of p<0.1. The contrast is overt picture naming over a rest period for all panels. Both deoxyHb and oxyHb results are represented in left and right columns, respectively. Clean, global-mean removed, and raw signals are shown in top and bottom rows, respectively. All conditions include left sagittal and dorsal views. Red-yellow indicates picture naming >rest and blue-green indicates rest>picture naming.

**Fig. 5:**
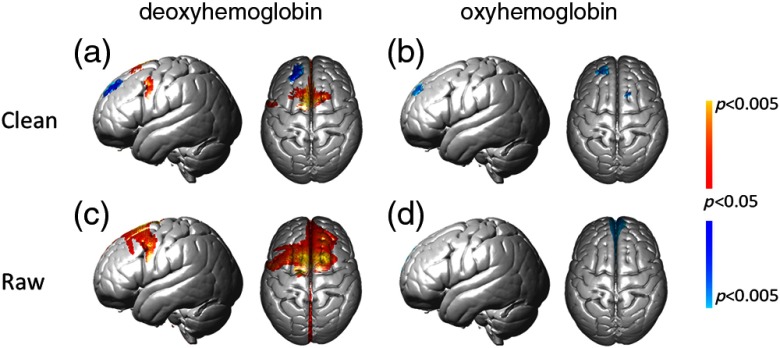
FDR corrected fNIRS results. fNIRS activity is shown with and without the global component removed at a corrected threshold of p<0.05, (FDR). The contrast is overt picture naming over rest period for all panels. Both deoxyHb and oxyHb results are represented in left and right columns, respectively. Clean, global-mean removed, and raw signals are shown in top and bottom rows, respectively. Views and color conventions are as described for Fig. 4.

We report results from both the deoxyHb and oxyHb signals that were processed (1) to remove global components (“clean” results) and (2) to show the unmodified signals (“raw” results). Figures 4(a) and 4(c) show the uncorrected results at a lenient threshold to illustrate the overall pattern of activity. The clean deoxyHb (upper left) data shows positive (red-yellow) activity covering left pars triangularis, premotor, and supplementary areas. While raw deoxyHb data show distributed activity covering most of the entire recorded area, data from deoxyHb signals with the application of the spatial filter were corrected for multiple comparison error using FDR (p<0.05),^30^ and are shown in Figs. 5(a) and 5(b) and Table 3 (Appendix C).

### 3.2 Oxyhemoglobin Results

Uncorrected and lenient results obtained from the oxyHb signals with and without the spatial filter are shown in Figs. 4(b) and 4(d) to illustrate the general distribution patterns. Both the clean and raw signals show a large cluster of negative activity covering most of the recording area. Negative activity indicates that the oxygen concentration was higher during baseline (resting) epochs compared to speaking epochs. Thresholded and corrected results from the spatially filtered oxyHb signal [Fig. 5(b)] showed a cluster of negative activity in dorsolateral prefrontal cortex with peak MNI coordinate (−18, 46, 36) (p≤0.05, FDR, t=−4.00). Corrected results from the raw oxyHb signal [Fig. 5(a)] showed a single cluster of negative activity in the frontopolar area with peak MNI coordinate (4, 60, 32) (p≤0.05, FDR, t=−3.91, n of voxels=36).

### 3.3 Event Triggered Average Results

**Fig. 6:**
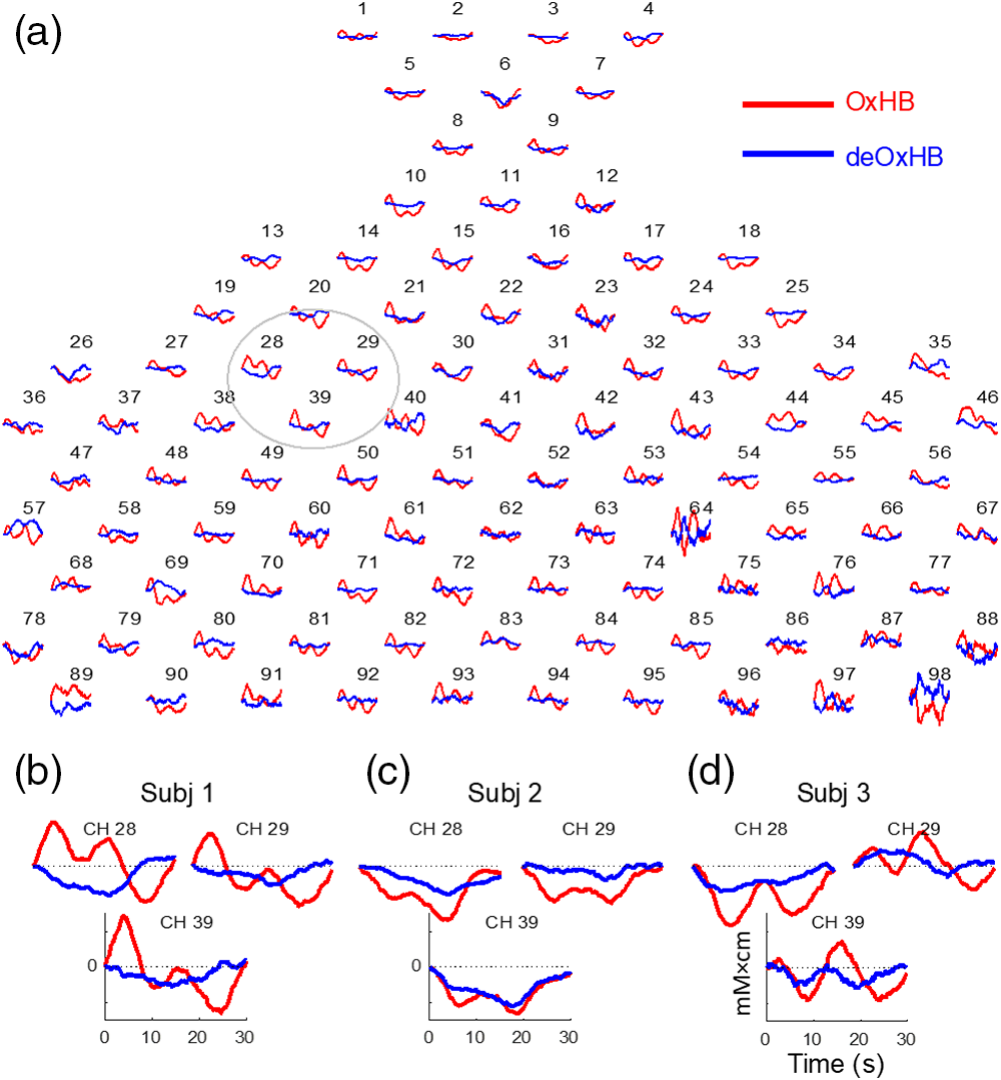
Event-triggered data prior to spatial filtering. (a) Event-triggered average plot showing all 98 channels in a representative subject. Data were averaged over the six 30-s task blocks. Red lines show oxyHb; blue lines show deoxyHb. (b)–(d) Data from three channels are enlarged with axis shown (same axis for all channels) from three individual subjects indicating variation in relative hemoglobin change profiles.

Figure 6(a) shows the event-triggered average plot for each channel from a representative subject prior to general linear modeling analyses. Following the fNIRS data presentation convention as stated above, both an upward oxyHb signal (red) and a downward deoxyHb signal (blue) indicate positive neural activity. A global component is clearly visible in all of the channels and is especially noticeable in the oxyHb (“w-shaped” signal). The oxyHb signal shows a decrease (negative activity) in almost all channels consistent with the raw data shown in Fig. 4(d). The deoxyHb signal shows a decrease (positive activity) in almost all channels, consistent with the raw data shown in Fig. 4(b). Figures 6(b)–6(d) show data from three channels [outlined in Fig. 6(a)] in three individual subjects that are enlarged to show additional local variation in the temporal aspects of the oxyHb signal contrasted with the deoxyHb signal.

### 3.4 Comparison of Functional NIRS, Neurosynth, and Functional Magnetic Resonance Imaging Results

**Fig. 7:**
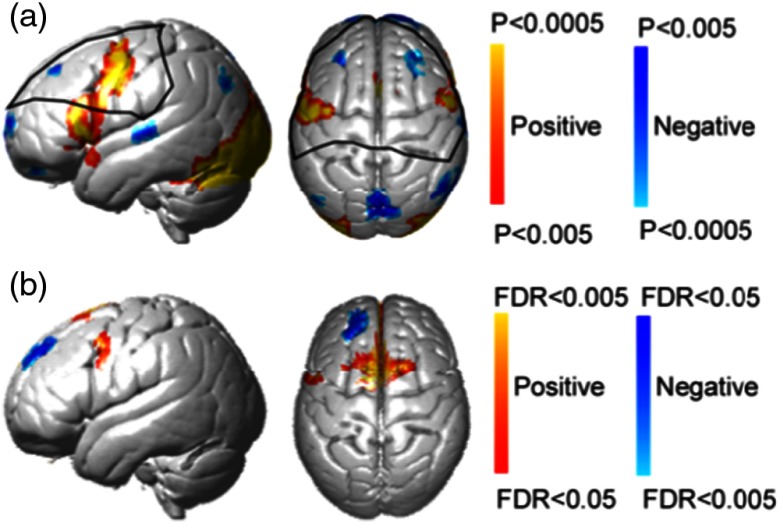
(a) fMRI activity for silent picture-naming task.^25^ (b) Voxel-wise analysis showing fNIRS activity for the overt picture-naming task measured with deoxyHb data after global component removal (p<0.05, corrected for multiple comparisons using FDR). The black lines delineate the voxels covered by all subjects in the fNIRS recording.

An independent fMRI dataset based on a similar task and paradigm is presented here for comparison with the fNIRS findings^7^^,^^25^ [Fig. 7(a)]. Although the task completed during acquisition of these fMRI images was covert (silent) naming rather than our overt (spoken) picture naming, the activity around Broca’s region is expected to be similar and serves as a second fiducial marker for the findings of this study. Figure 7(b) shows the neural activity measured with fNIRS deoxyHb data after global component removal. Within the coverage of the fNIRS channels, activity around Broca’s region overlays the activity shown in the fMRI data. Note that the optode coverage [Fig. 2(a)] does not include the most lateral ventral regions observed in either the fMRI data [Fig. 7(a)] or the Neurosynth marker (Fig. 1). The fNIRS data [Fig. 7(b), dorsal view] show increased activity near the supplementary motor area (SMA) as compared to the fMRI data [Fig. 7(a), dorsal view]. This is as expected for an overt speaking task where the supplementary motor system is actively engaged during speech articulation.

**Fig. 8:**
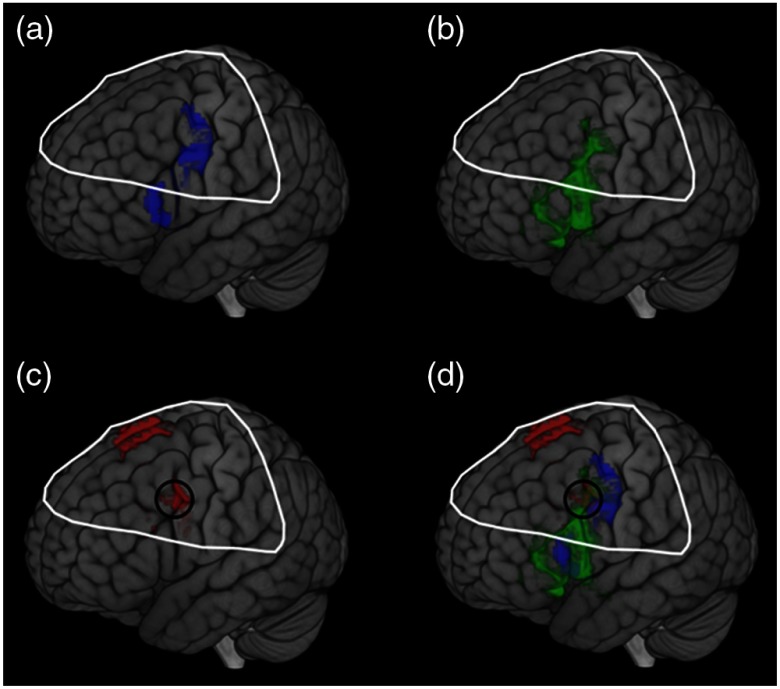
(a) fMRI activity for silent picture-naming task.^25^ (b) fMRI activity for Neurosynth data (search terms: “Broca”). (c) Voxel-wise fNIRS activity for the overt picture-naming task measured with deoxyHb signal after global component removal (p<0.05, FDR corrected). (d) Synthesis of activation data during speech tasks from (a) to (c). The white line surrounds the area of fNIRS coverage (all subjects) and the black circle shows the cluster of fNIRS activity within the area of overlap between all three methods.

The result obtained from the spatially filtered deoxyHb signals was compared with the fMRI data set, Fig. 1(a), and the Neurosynth map of Broca’s area (Fig. 1). Figure 8 shows the fMRI activity during covert speaking [Fig. 8(a)], the Neurosynth map of Broca’s area [Fig. 8(b)], and the present fNIRS result [Fig. 8(d)]. The overlap of all three is shown within the open circle in Fig. 8(c), illustrating a common area of activity. Note that since SPM group analysis is limited to the channels that are present for all subjects, the fNIRS coverage shown in Fig. 8 (the white boundary) is smaller than the individual coverage shown as median channel locations in Fig. 2(a). As shown in Fig. 8, the coverage in common across all subjects does not include the most ventral regions observed in either the fMRI data [Fig. 8(a)] or the Neurosynth marker [Fig. 8(b)].

## 4 Discussion

Previously, we have shown that global component removal during preprocessing using spatial filtering reveals activity consistent with expected cortical activity for finger tapping tasks.^21^ Here, we extend these findings to include overt speaking and determine that this spatial filter can be applied for deoxyHb signals, revealing expected cortical activity in areas of the brain specialized for speech production. Specifically, “clean” deoxyHb signals yielded activity localized to left frontal regions included in Broca’s region, and pre- and supplementary motor cortex consistent with a previous fMRI study using a similar task and paradigm with silent speech^25^ as well as the Neurosynth meta-analysis using a wide range of silent language tasks performed during scanning with fMRI. Both are consistent with well-described findings from intraoperative stimulation.

Although the deoxyHb signals with global component removal show specific activity in Broca’s region and the SMA [Fig. 5(a)], the unfiltered deoxyHb data show widespread global component [Figs. 5(c)] during the picture-naming task. This is different from our previous findings based on finger thumb tapping, which suggested that global components in the deoxyHb were not significant.^21^ The current results imply that the global component in the deoxyHb signal is more apparent in some tasks than others, suggesting that global component removal is generally beneficial to an analysis pipeline to maximize the likelihood of reflecting neural activity.

The coupling between neurological and physiological processes that underlie changes in oxyHb and deoxyHb concentrations in the brain during cognitive and motor tasks is an active topic of investigation. The anticorrelation between these two signals that is typically observed during task-rest cycles is believed to reflect (1) increases in blood flow related to neutrally active tissue and serves as a proxy for task-specific neural activity that underlies cognitive function; (2) increases in blood flow related to systemic physiological factors; and (3) relative decreases in deoxyHb concentrations also related to neurovascular coupling and serves as a proxy for neural activity, respectively. Multiple systemic physiological factors not directly related to the neurovascular coupling have been described.18 For example, variations in partial pressure of end-tidal carbon dioxide (${\mathrm{PetCO}}_{2}$) associated with respiration have been observed during speech production and shown to decrease with similar tasks performed with only internal and cognitive responses.^20^ Other nonneural physiological factors, such as heart rate, blood pressure, respiration rate, and concentration of ${\mathrm{CO}}_{2}$, have also been shown to influence blood oxygen concentrations as measured by fNIRS (Refs. 18, 31 and 32). It is widely understood that these factors are modulated by subject characteristics, such as age, gender, fitness, body size, time from exercise, medications, anxiety levels, and further complicate computational approaches to separate neural and systemic components in both oxyHb and deoxyHb signals. Furthermore, assumptions of equal variance across whole brains of individual subjects may also be violated by both individual differences and task demands.^33^ To the extent that these sources of variation are systemic in origin, they would be expected to differentially affect the oxyHb and deoxyHb signals. For example, the task related increase in the oxyHb signal is attributed to both neural and systemic physiological factors, whereas the task-related decrease in the deoxyHb signal is primarily attributed to neurovascular coupling.

The paradoxical group observation in the unthresholded, averaged raw oxyHb signals [Fig. 4(d)], showing both the absence of signal in the ROI, Broca’s Area, and the negative group average in frontal areas is consistent with the hypothesis that systemic factors such as end-tidal carbon dioxide may have resulted in a negative signal. Regional differences in systemic factors were also present, as illustrated by the difference between the oxyHb signal in the three channels in Figs. 6(b)–6(d). These localized systemic effects may have prevented the spatial filter from adequately removing this global negative signal, as shown by the group-averaged result in Fig. 5(b). When the oxyHb was subjected to a threshold and multiple comparisons correction, individual differences in systemic factors may have washed out a group effect. However, the widely distributed group signal for the simultaneously acquired raw deoxyHb data, Fig. 4(c), suggests that the deoxyHb signal may be less affected by these sources of variation than the oxyHb signal for a speaking task. This suggestion and observation is an important topic for future research and the development of computational and experimental approaches as fNIRS emerges as a method of choice for studies of cognitive processes in natural conditions.

## 5 Limitations

The finding that group data for the oxyHb signal during the overt speaking did not reveal canonical regions associated with Broca’s area, i.e., left pre- and supplementary motor cortex and left pars opercularis, was unexpected. Although increased individual variability of systemic factors associated with breathing that occur during a speaking task as well as individually specific regional brain differences may contribute, there are other possible contributing factors. The movement of head, mouth, and the temporalis muscle during overt speech creates particularly challenging circumstances for an imaging study. These findings suggest that future investigations of speech functions would benefit from movement extraction algorithms, and, in particular, the oxyHb signal may benefit from simultaneous measurements of ${\mathrm{PetCO}}_{2}$, as previously suggested by Scholkmann et al.^20^ Algorithms that employ physiological regressors to further refine the separation between neural and systemic effects, in addition to ${\mathrm{PetCO}}_{2}$, such as heart rate, blood pressure, and respiration,^18^ may also be particularly beneficial to the oxyHb signal. Additionally, while traditional short channel regression techniques in the temporal domain may also remove cortical responses, newer techniques that only regress data that only has a positive (nonstandard) correlation between oxyHb and deoxyHb have been suggested and may further increase signal to noise in the oxyHb recordings.^33^

An additional limitation of the study was the variability of detector locations in the inferior aspect of the left frontal lobe. This was due to the effects of variability of channel location in that area resulting from variations in head and cap size. As the field of view indicates (Fig. 3), the inferior aspects of Broca’s area were not reliably sampled. This is a potential pitfall that can be avoided in future investigations with cap sizes designed to fit various head sizes.

## 6 Conclusion

In this study, we compared fNIRS activity from an overt picture-naming task to both a Neurosynth activity map and fMRI activity during a silent picture-naming task.^25^ Spatial filtering of global components from the fNIRS deoxyHb signal yielded results similar to those obtained with fMRI. Even after spatial filtering, fNIRS oxyHb signals did not show expected activity patterns related to picture naming. One possible explanation is that the oxyHb signal is more sensitive to modulation by systemic sources. The deoxyHb yielded activity patterns similar to fMRI and Neurosynth results only after global component removal was applied. This study is the first to our knowledge to show the benefits of systemic artifact removal on fNIRS signals recorded during a task involving spoken language to eliminate neural responses from Broca’s area. Findings suggest that fNIRS may be used to study spoken language outside the confines of an fMRI scanner and thereby extends the applications of fNIRS to neuroimaging in natural and freely moving conditions.

## Appendix A: Median Channel Locations

The median locations for each channel are listed in Table 1.

**Table 1:** Median channel locations for all subjects. The X, Y, and Z columns represent MNI coordinates. MNI coordinates were converted to Talairach coordinates to generate anatomical areas. The last column lists the atlas-based probability that the XYZ coordinates are within that anatomical location (only probabilities greater than 20% were listed here).

Channel	X	Y	Z	BA-anatomy	Probability
1	−32	63	15	10-frontopolar area	1
2	−12	68	23	10-frontopolar area	1
3	15	68	24	10-frontopolar area	1
4	34	63	18	10-frontopolar area	1
5	−20	62	30	9-dorsolateral prefrontal cortex	0.3
				10-frontopolar area	0.7
6	2	61	34	9-dorsolateral prefrontal cortex	0.49
				10-frontopolar area	0.51
7	22	61	32	9-dorsolateral prefrontal cortex	0.42
				10-frontopolar area	0.58
8	−11	57	42	9-dorsolateral prefrontal cortex	0.84
9	13	56	42	8-includes Frontal eye fields	0.21
				9-dorsolateral prefrontal cortex	0.79
10	−19	48	47	8-includes frontal eye fields	0.69
				9-dorsolateral prefrontal cortex	0.31
11	1	48	49	8-includes frontal eye fields	0.85
12	20	48	48	8-Includes frontal eye fields	0.81
13	−44	40	33	9-dorsolateral prefrontal cortex	0.39
				46-dorsolateral prefrontal cortex	0.6
14	−28	39	48	8-includes frontal eye fields	0.85
15	−11	41	56	8-includes frontal eye fields	0.95
16	13	41	57	8-includes frontal eye fields	0.92
17	26	39	50	8-includes frontal eye fields	1
				9-dorsolateral prefrontal cortex	0.69
19	−51	29	32	46-dorsolateral prefrontal cortex	0.57
20	−39	28	49	8-includes frontal eye fields	0.94
21	−19	30	60	6-premotor and supplementary motor cortex	0.49
				8-includes frontal eye fields	0.51
22	0	31	60	6-premotor and supplementary motor cortex	0.52
				8-includes frontal eye fields	0.48
23	20	31	61	6-premotor and supplementary motor cortex	0.54
				8-includes frontal eye fields	0.46
24	37	28	52	8-includes frontal eye fields	1
25	52	30	35	9-dorsolateral prefrontal cortex	0.6
				46-dorsolateral prefrontal cortex	0.4
26	−60	16	7	44-pars opercularis, part of Broca’s area	0.41
				45-pars triangularis Broca’s area	0.33
27	−57	18	29	9-dorsolateral prefrontal cortex	0.66
				45-pars triangularis Broca’s area	0.23
28	−46	20	49	8-includes frontal eye fields	0.82
29	−31	20	61	6-premotor and supplementary motor cortex	0.53
				8-includes frontal eye fields	0.47
30	−13	22	67	6-premotor and supplementary motor cortex	1
31	13	22	67	6-premotor and supplementary motor cortex	1
32	31	21	62	6-premotor and supplementary motor cortex	0.66
				8-includes frontal eye fields	0.34
33	47	20	51	8-includes frontal eye fields	0.87
34	58	18	33	9-dorsolateral prefrontal cortex	0.85
35	62	16	11	45-pars triangularis Broca’s area	0.41
				44-pars opercularis, part of Broca’s area	0.54
36	−65	−1	−5	21-middle temporal gyrus	0.64
				22-superior temporal gyrus	0.35
37	−63	4	25	6-premotor and supplementary motor cortex	0.63
38	−54	7	45	6-premotor and supplementary motor cortex	0.57
				8-includes frontal eye fields	0.21
				9-dorsolateral prefrontal cortex	0.22
39	−41	12	60	6-premotor and supplementary motor cortex	0.73
				8-includes frontal eye fields	0.27
40	−21	12	69	6-premotor and supplementary motor cortex	1
41	−1	11	70	6-premotor and supplementary motor cortex	1
42	21	10	71	6-premotor and supplementary motor cortex	1
43	40	10	61	6-premotor and supplementary motor cortex	0.86
44	55	7	48	6-premotor and supplementary motor cortex	0.67
				8-includes frontal eye fields	0.22
45	65	5	28	6-premotor and supplementary motor cortex	0.6
				9-dorsolateral prefrontal cortex	0.31
46	67	1	1	21-middle temporal gyrus	0.32
				22-superior temporal gyrus	0.59
47	−67	−8	17	43-subcentral area	0.42
48	−62	−5	39	6-premotor and supplementary motor cortex	0.98
49	−49	−1	56	6-premotor and supplementary motor cortex	0.93
50	−31	1	68	6-premotor and supplementary motor cortex	1
51	−13	0	75	6-premotor and supplementary motor cortex	1
52	13	0	75	6-premotor and supplementary motor cortex	1
53	30	−2	70	6-premotor and supplementary motor cortex	1
54	49	−3	59	6-premotor and supplementary motor cortex	0.9
55	62	−5	43	6-premotor and supplementary motor cortex	0.95
56	69	−7	20	6-premotor and supplementary motor cortex	0.32
				43-subcentral area	0.4
57	−70	−22	0	21-middle temporal gyrus	0.48
				22-superior temporal gyrus	0.32
				42-primary and auditory association cortex	0.2
58	−67	−19	29	2-primary somatosensory cortex	0.24
59	−59	−16	49	3-primary somatosensory cortex	0.23
				6-premotor and supplementary motor cortex	0.36
60	−43	−13	65	6-premotor and supplementary motor cortex	0.72
61	−22	−12	75	6-premotor and supplementary motor cortex	1
62	−2	−11	75	6-premotor and supplementary motor cortex	1
63	22	−12	76	6-premotor and supplementary motor cortex	1
64	42	−15	68	6-premotor and supplementary motor cortex	0.75
65	58	−18	53	3-primary somatosensory cortex	0.39
66	68	−20	33	1-primary somatosensory cortex	0.25
				2-primary somatosensory cortex	0.22
67	72	−22	4	21-middle temporal gyrus	0.22
				22-superior temporal gyrus	0.41
				42-primary and auditory association cortex	0.37
68	−69	−33	14	22-superior temporal gyrus	0.57
				40-supramarginal gyrus part of Wernicke’s area	0.09
				42-primary and auditory association cortex	0.34
69	−65	−30	39	2-primary somatosensory cortex	0.21
				40-supramarginal gyrus part of Wernicke’s area	0.61
70	−53	−26	59	1-primary somatosensory cortex	0.26
				2-primary somatosensory cortex	0.39
				3-primary somatosensory cortex	0.2
71	−35	−23	72	4-primary motor cortex	0.31
				6-premotor and supplementary motor cortex	0.52
72	−13	−23	79	4-primary motor cortex	0.28
				6-premotor and supplementary motor cortex	0.72
73	13	−24	79	4-primary motor cortex	0.24
				6-premotor and supplementary motor cortex	0.76
74	33	−24	73	4-primary motor cortex	0.43
				6-premotor and supplementary motor cortex	0.49
75	52	−27	62	1-primary somatosensory cortex	0.28
				2-primary somatosensory cortex	0.23
				3-primary somatosensory cortex	0.33
76	65	−31	44	2-primary somatosensory cortex	0.2
				40-supramarginal gyrus part of Wernicke’s area	0.59
77	71	−34	18	22-superior temporal gyrus	0.44
				40-supramarginal gyrus part of Wernicke’s area	0.29
				42-primary and auditory association cortex	0.27
78	−68	−45	−3	21-middle temporal gyrus	0.79
79	−67	−43	24	22-superior temporal gyrus	0.37
				40-supramarginal gyrus part of Wernicke’s area	0.63
80	−61	−40	46	40-supramarginal gyrus part of Wernicke’s area	0.98
81	−46	−35	64	1-primary somatosensory cortex	0.21
				2-primary somatosensory cortex	0.33
				40-supramarginal gyrus part of Wernicke’s area	0.31
82	−24	−36	75	3-primary somatosensory cortex	0.39
				4-primary motor cortex	0.24
83	−1	−37	78	4-primary motor cortex	0.33
				6-premotor and supplementary motor cortex	0.45
84	22	−38	77	3-primary somatosensory cortex	0.45
				4-primary motor cortex	0.26
85	44	−37	66	2-primary somatosensory cortex	0.3
86	59	−42	50	40-supramarginal gyrus part of Wernicke’s area	1
87	67	−46	27	40-supramarginal gyrus part of Wernicke’s area	0.81
88	69	−47	−2	21-middle temporal gyrus	0.63
				22-superior temporal gyrus	0.22
89	−64	−55	5	21-middle temporal gyrus	0.66
				22-superior temporal gyrus	0.24
90	−62	−54	31	40-supramarginal gyrus part of Wernicke’s area	0.81
91	−53	−51	52	40-supramarginal gyrus part of Wernicke’s area	1
92	−36	−49	68	5-somatosensory association cortex	0.51
				7-somatosensory association cortex	0.26
93	−14	−50	76	5-somatosensory association cortex	0.36
				7-somatosensory association cortex	0.51
94	13	−50	76	5-somatosensory association cortex	0.37
				7-somatosensory association cortex	0.5
95	33	−51	69	5-somatosensory association cortex	0.45
				7-somatosensory association cortex	0.54
96	50	−54	55	40-supramarginal gyrus part of Wernicke’s area	0.93
97	60	−58	34	39-angular gyrus, part of Wernicke’s area	0.32
				40-supramarginal gyrus part of Wernicke’s area	0.68
98	64	−60	7	21-middle temporal gyrus	0.45
				22-superior temporal gyrus	0.21

## Appendix B: Optimization of the Global Component Removal Method

The PCA global component removal method is essentially a spatial high-pass Gaussian filter method.^21^ The following equation described the Gaussian filter:

$$G_{2-D}(r)=\exp(-{\text{distance}}^{2}/2\sigma_{2}),$$

where σ represents the width at half-maximum of the Gaussian kernel. In the previous paper, we set the parameter σ as 50 deg based on the observed extent of global component, noting that this value should be greater than the width of the expected cortical activation but smaller than the width of the global components. To optimize this width, we used data from 22 participants performing a right-handed finger-tapping task. Data were averaged across 3×3×3 voxels (each $\text{voxel}=2\;{\mathrm{mm}}^{3}$) in the left motor cortex. We tested different values for σ and calculated the peak T value for each σ, as shown in Table 2.

**Table 2:** Peak T values for each angle (σ).

σ (deg)	T
42.5	2.57
44.4	2.61
46.2	2.62
48.0	2.51
50.0	2.52
51.2	2.53
52.8	2.56

Using this procedure, we found that the value for the filter parameter σ that optimized the peak T value of motor cortex activity was 46 deg instead of the previously adopted value of 50 deg. Because of this, we used a parameter value σ of 46 deg in this study.

## Appendix C: Voxel-Wise and Channel-Wise Results from Clean deoxyHb Signals

The results of the corrected voxel-wise analyses of the deoxyHb (Table 3) and oxyHb (Table 4) signals with the spatial filter (“clean”) are reported.

**Table 3:** Contrast comparisons (deoxyHb signals, clean, FDR corrected) for voxel-wise analysis.

Contrast	Contrast threshold (FDR adjusted)	Peak Voxel				Anatomical regions in cluster	BA^b^	Anatomical probability
		MINI Coordinate^a^			t value			
[Picture-naming>rest]	p=0.05	−56	6	30	3.48	Pre- and supplementary motor cortex	6	0.70
						Pars opercularis, part of Broca’s area	44	0.22
						Primary motor cortex	4	0.04
						Subcentral area	43	0.04

a Coordinates are based on the MNI system and (−) indicates left hemisphere.

b BA, Brodmann area.

**Table 4:** Contrast comparisons (oxyHb signals, clean, FDR corrected) for voxel-wise analysis.

Contrast	Contrast threshold (FDR adjusted)	Peak Voxel				Anatomical regions in cluster	BA^b^	Anatomical probability
		MINI Coordinate^a^			t value			
[Picture-naming>rest]	p=0.05	−18	46	36	−4.00	Dorsolateral prefrontal cortex	9	0.70
						Frontal eye fields	8	0.30

a Coordinates are based on the MNI system and (−) indicates left hemisphere.

b BA, Brodmann area.

## References

[1] Price C. J., “A review and synthesis of the first 20 years of PET and fMRI studies of heard speech, spoken language and reading,” NeuroImage 62(2), 816–847 (2012). NEIMEF 1053-8119 10.1016/j.neuroimage.2012.04.062 22584224 PMC3398395

[2] Edmister W. B. et al., “Improved auditory cortex imaging using clustered volume acquisitions,” Hum. Brain Mapp. 7(2), 89–97 (1999). HBRME7 1065-9471 10.1002/(ISSN)1097-0193 9950066 PMC6873308

[3] Hall D. A. et al., ““Sparse” temporal sampling in auditory fMRI,” Hum. Brain Mapp. 7(3), 213–223 (1999). HBRME7 1065-9471 10.1002/(ISSN)1097-0193 10194620 PMC6873323

[4] Abel T. J. et al., “Direct physiologic evidence of a heteromodal convergence region for proper naming in human left anterior temporal lobe,” J. Neurosci. 35(4), 1513–1520 (2015). JNRSDS 0270-6474 10.1523/JNEUROSCI.3387-14.2015 25632128 PMC4308598

[r5] Hagoort P., “Nodes and networks in the neural architecture for language: Broca’s region and beyond,” Curr. Opin. Neurobiol. 28, 136–141 (2014). COPUEN 0959-4388 10.1016/j.conb.2014.07.013 25062474

[6] Poeppel D., “The neuroanatomic and neurophysiological infrastructure for speech and language,” Curr. Opin. Neurobiol. 28, 142–149 (2014). COPUEN 0959-4388 10.1016/j.conb.2014.07.005 25064048 PMC4177440

[7] Hirsch J. et al., “An integrated functional magnetic resonance imaging procedure for preoperative mapping of cortical areas associated with tactile, motor, language, and visual functions,” Neurosurgery 47(3), 711–722 (2000). NEQUEB 10.1097/00006123-200009000-00037 10981759

[8] Yarkoni T. et al., “Large-scale automated synthesis of human functional neuroimaging data,” Nat. Methods 8(8), 665–670 (2011).1548-7091 10.1038/nmeth.1635 21706013 PMC3146590

[9] Scholkmann F. et al., “A review on continuous wave functional near-infrared spectroscopy and imaging instrumentation and methodology,” NeuroImage 85 (Pt. 1), 6–27 (2014). NEIMEF 1053-8119 10.1016/j.neuroimage.2013.05.004 23684868

[10] Ferrari M. Quaresima V., “A brief review on the history of human functional near-infrared spectroscopy (fNIRS) development and fields of application,” NeuroImage 63(2), 921–935 (2012). NEIMEF 1053-8119 10.1016/j.neuroimage.2012.03.049 22510258

[r11] Franceschini M. A. Boas D. A., “Noninvasive measurement of neuronal activity with near-infrared optical imaging,” NeuroImage 21(1), 372–386 (2004). NEIMEF 1053-8119 10.1016/j.neuroimage.2003.09.040 14741675 PMC3786741

[r12] Cui X. et al., “A quantitative comparison of NIRS and fMRI across multiple cognitive tasks,” NeuroImage 54(4), 2808–2821 (2011). NEIMEF 1053-8119 10.1016/j.neuroimage.2010.10.069 21047559 PMC3021967

[r13] Ferrari M. Mottola L. Quaresima V., “Principles, techniques, and limitations of near infrared spectroscopy,” Can. J. Appl. Physiol. 29(4), 463–487 (2004). 10.1139/h04-031 15328595

[r14] Strangman G. et al., “A quantitative comparison of simultaneous BOLD fMRI and NIRS recordings during functional brain activation,” NeuroImage 17(2), 719–731 (2002). NEIMEF 1053-8119 10.1006/nimg.2002.1227 12377147

[15] Villringer A. Chance B., “Non-invasive optical spectroscopy and imaging of human brain function,” Trends Neurosci. 20(10), 435–442 (1997). TNSCDR 0166-2236 10.1016/S0166-2236(97)01132-6 9347608

[16] Kirilina E. et al., “The physiological origin of task-evoked systemic artefacts in functional near infrared spectroscopy,” NeuroImage 61(1), 70–81 (2012). NEIMEF 1053-8119 10.1016/j.neuroimage.2012.02.074 22426347 PMC3348501

[r17] Boas D. A. et al., “Twenty years of functional near-infrared spectroscopy: introduction for the special issue,” NeuroImage 85(Pt. 1), 1–5 (2014). NEIMEF 1053-8119 10.1016/j.neuroimage.2013.11.033 24321364

[18] Tachtsidis I. Scholkmann F., “False positives and false negatives in functional near-infrared spectroscopy: issues, challenges, and the way forward,” Neurophotonics 3(3), 031405 (2016). 10.1117/1.NPh.3.3.031405 27054143 PMC4791590

[19] Boas D. A. Dale A. M. Franceschini M. A., “Diffuse optical imaging of brain activation: approaches to optimizing image sensitivity, resolution, and accuracy,” NeuroImage 23(Suppl. 1), S275–S288 (2004). NEIMEF 1053-8119 10.1016/j.neuroimage.2004.07.011 15501097

[20] Scholkmann F. et al., “End-tidal ${\mathrm{CO}}_{2}$: an important parameter for a correct interpretation in functional brain studies using speech tasks,” NeuroImage 66, 71–79 (2013). NEIMEF 1053-8119 10.1016/j.neuroimage.2012.10.025 23099101

[21] Zhang X. Noah J. A. Hirsch J., “Separation of the global and local components in functional near-infrared spectroscopy signals using principal component spatial filtering,” Neurophotonics 3(1), 015004 (2016). 10.1117/1.NPh.3.1.015004 26866047 PMC4742567

[22] Funane T. et al., “Quantitative evaluation of deep and shallow tissue layers’ contribution to fNIRS signal using multi-distance optodes and independent component analysis,” NeuroImage 85(Pt. 1), 150–165 (2014). NEIMEF 1053-8119 10.1016/j.neuroimage.2013.02.026 23439443

[23] Gagnon L. et al., “Further improvement in reducing superficial contamination in NIRS using double short separation measurements,” NeuroImage 85, 127–135 (2014). NEIMEF 1053-8119 10.1016/j.neuroimage.2013.01.073 23403181 PMC3665655

[24] Kaplan E. Goodglass H. Weintraub S., Boston Naming Test, Pro-ed, Austin (2001).

[25] Hirsch J. Moreno D. R. Kim K. H., “Interconnected large-scale systems for three fundamental cognitive tasks revealed by functional MRI,” J. Cognit. Neurosci. 13(3), 389–405 (2001). JCONEO 0898-929X 10.1162/08989290151137421 11371315

[26] Oldfield R. C., “The assessment and analysis of handedness: the Edinburgh inventory,” Neuropsychologia 9(1), 97–113 (1971). NUPSA6 0028-3932 10.1016/0028-3932(71)90067-4 5146491

[27] Jasper H. H., “Report of the committee on methods of clinical examination in electroencephalography: 1957,” Electroencephalogr. Clin. Neurophysiol. 10(2), 370–375 (1958). 10.1016/0013-4694(58)90053-1

[28] Ye J. C. et al., “NIRS-SPM: statistical parametric mapping for near-infrared spectroscopy,” NeuroImage 44(2), 428–447 (2009). NEIMEF 1053-8119 10.1016/j.neuroimage.2008.08.036 18848897

[29] Friston K. et al., “Statistical parametric maps in functional imaging: a general linear approach,” Hum. Brain Mapp. 2, 189–210 (1994). HBRME7 1065-9471 10.1002/hbm.v2:4

[30] Benjamini Y. Hochberg Y., “Controlling the false discovery rate—a practical and powerful approach to multiple testing,” J. R. Stat. Soc. Ser. B Method. 57(1), 289–300 (1995).0952-8385 10.2307/2346101

[31] Yamada T. Umeyama S. Matsuda K., “Separation of fNIRS signals into functional and systemic components based on differences in hemodynamic modalities,” PLoS One 7(11): e50271 (2012). 10.1371/journal.pone.0050271 23185590 PMC3501470

[32] Moody M. et al., “Cerebral and systemic hemodynamic changes during cognitive and motor activation paradigms,” Am. J. Physiol.-Regul. Integr. Comp. Physiol. 288(6), R1581–R1588 (2005). 10.1152/ajpregu.00837.2004 15677522

[33] Yamamoto T. Kato T., “Paradoxical correlation between signal in functional magnetic resonance imaging and deoxygenated haemoglobin content in capillaries: a new theoretical explanation,” Phys. Med. Biol. 47(7), 1121–1141 (2002). PHMBA7 0031-9155 10.1088/0031-9155/47/7/309 11996059

